# Increased spectral power of theta rhythm is not associated with decreased supragranular thickness in first-episode schizophrenia

**DOI:** 10.1192/j.eurpsy.2022.847

**Published:** 2022-09-01

**Authors:** A. Tomyshev, I. Lebedeva, E. Abdullina, V. Kaleda

**Affiliations:** 1 Mental Health Research Center, Laboratory Of Neuroimaging And Multimodal Analysis, Moscow, Russian Federation; 2 Mental Health Research Center, Department Of Youth Psychiatry, Moscow, Russian Federation

**Keywords:** MRI, schizophrénia, Theta rhythm, Supragranular thinning

## Abstract

**Introduction:**

Schizophrenia is associated with disturbances in neurophysiological processes. However, the relation of EEG and ERP parameters to structural supragranular cortical abnormalities, observed in schizophrenia, remains unclear.

**Objectives:**

The purpose was to characterize EEG and ERP disturbances and their relationship to changes occurring in supragranular cortical layers in subjects with schizophrenia.

**Methods:**

43 first-episode schizophrenia (FES) male patients and 43 matched healthy controls (HC) underwent background EEG and standard two-tones oddball ERP recording and structural MRI at 3T Philips scanner. MRI images were processed via FreeSurfer and MATLAB to derive two markers specific to supragranular thickness change: gyral-sulcal thickness differences (GSTD) and gyral-sulcal intrinsic curvature differences on pial surface (GSCD) (github.com/kwagstyl/schizophrenia_gyral_sulcal).

**Results:**

Theta rhythm spectral power was increased in FES while P300 amplitudes and latencies, N100 (to non-targets) amplitudes, alpha rhythm spectral power were not altered compared to HC. GSCD measures were increased in temporal, parietal and occipital cortices, whereas both GSTD and GSCD were increased in the right frontal cortex in FES. No correlations between altered EEG and supragranular thickness markers survived correction for multiple comparisons.

**Conclusions:**

Presumably, theta rhythm has a widespread circuit of generators, including the cortical ones. However, we have not found correlations between EEG and supragranular markers in FES. Considering an absence of correlations between theta and hippocampal volumes (Lebedeva et al., 2020), a speculative interpretation is that the neurophysiological disturbances may be associated with a more complex patterns of more localized structural and functional impairments.

**Disclosure:**

The work was supported by RFBR grant 20-013-00748.

